# Sensorimotor, Cognitive and Affective Barriers to Social Relations in Aging: A Dynamical Framework

**DOI:** 10.1093/geroni/igab046.3331

**Published:** 2021-12-17

**Authors:** Emmanuelle Tognoli, Alice Wead, Joseph McKinley, Christopher Beetle, Christine Williams

**Affiliations:** 1 Florida Atlantic University, Boca Raton, Florida, United States; 2 Florida Atlantic University, BOCA RATON, Florida, United States

## Abstract

Social interactions of all sorts (e.g. conversing, playing tennis, singing, strolling, etc.) rely on information flows between participants. The process of aging, however, can alter individuals’ sensorial, motor, cognitive and affective functioning in ways that may compromise their affinity for social interactions. For instance, hearing deficits or cognitive difficulties associated with word retrieval may contribute to disengagement from conversation and other forms of social interaction, which can lead to social retreat of the affected individuals. Strategies for mitigating such effects must take into account not only individuals’ own functional capacities, but also those of their partners in varying social contexts. Indeed, varied social contexts and diversity in partners can offer a beneficial balance of relational effort and comfort. For example, instead of comfortably strolling exclusively with partners of comparable cognitive and motor capabilities, strolling with faster partners can improve social engagement and long-term prospects for a wider range of social interactions. This work reviews an array of possible changes in individual abilities arising from both normal healthy aging and complications due to medical conditions, with an emphasis on their impact on interactions in varying social contexts and diverse groups of social partners. We incorporate theoretical models to explore a wide range of potential mitigation strategies, both for affected individuals and for other members of the social groups surrounding them. Our work focuses on healthy social aging over the long term, which is known to protect physical wellbeing, cognition and brain function.

